# Oleoylethanolamide restores stress-induced prepulse inhibition deficits and modulates inflammatory signaling in a sex-dependent manner

**DOI:** 10.1007/s00213-023-06403-w

**Published:** 2023-06-14

**Authors:** Macarena González-Portilla, Sandra Montagud-Romero, Fernando Rodríguez de Fonseca, Marta Rodríguez-Arias

**Affiliations:** 1 Department of Psychobiology, Faculty of Psychology, Universitat de València, Avda. Blasco Ibáñez 21, 46010 Valencia, Spain; 2 Unidad Clínica de Neurología, Instituto de Investigación Biomédica de Málaga-IBIMA, Hospital Regional Universitario de Málaga, 29010 Málaga, Spain; 3 Atención Primaria, Cronicidad Y Promoción de La Salud. Red de Investigación en Atención Primaria de Adicciones (RIAPAD) Rd21/0009/0005, Málaga, Spain

**Keywords:** Oleoylethanolamide, Stress, Social defeat, Prepulse inhibition, Cytokine, Neuroinflammation

## Abstract

**Rationale:**

Social stress contributes to the development of depressive and anxiety symptomatology and promotes pro-inflammatory signaling in the central nervous system. In this study, we explored the effects of a lipid messenger with anti-inflammatory properties – oleoylethanolamide (OEA) – on the behavioral deficits caused by social stress in both male and female mice.

**Methods:**

Adult mice were assigned to an experimental group according to the stress condition (control or stress) and treatment (vehicle or OEA, 10 mg/kg, i.p.). Male mice in the stress condition underwent a protocol consisting of four social defeat (SD) encounters. In the case of female mice, we employed a procedure of vicarious SD. After the stress protocol resumed, anxiety, depressive-like behavior, social interaction, and prepulse inhibition (PPI) were assessed. In addition, we characterized the stress-induced inflammatory profile by measuring IL-6 and CX3CL1 levels in the striatum and hippocampus.

**Results:**

Our results showed that both SD and VSD induced behavioral alterations. We found that OEA treatment restored PPI deficits in socially defeated mice. Also, OEA affected differently stress-induced anxiety and depressive-like behavior in male and female mice. Biochemical analyses showed that both male and female stressed mice showed increased levels of IL-6 in the striatum compared to control mice. Similarly, VSD female mice exhibited increased striatal CX3CL1 levels. These neuroinflammation-associated signals were not affected by OEA treatment.

**Conclusions:**

In summary, our results confirm that SD and VSD induced behavioral deficits together with inflammatory signaling in the striatum and hippocampus. We observed that OEA treatment reverses stress-induced PPI alterations in male and female mice. These data suggest that OEA can exert a buffering effect on stress-related sensorimotor gating behavioral processing.

## Introduction

Stress has long been identified as a key factor for the development, course, and treatment of mental disorders. Stress-related symptomatology constitutes a trans-diagnostic feature across a wide variety of psychiatric illnesses including anxiety disorder, bipolar disorder, substance use disorder, eating disorders, and schizophrenia (Lupien et al. 2009). The experience of excessive or prolonged psychosocial stress has been associated with disturbed emotional behavior and an increased vulnerability to clinical conditions such as anxiety and depression (Cohen et al. 2012; McEwen 2004). Low prepulse inhibition (PPI) of the acoustic reflex has been proposed as an endophenotype associated with an increased risk for multiple psychiatric conditions (Braff et al. 2001; Kohl et al. 2013). Preclinical studies have shown that several stress challenges such as social isolation, restraint stress, or maternal separation induce PPI deficits (Arenas et al. 2022; Chen et al. 2010; Dai et al. 2004; Ellenbroek et al. 1998).

In the past decade, there has been compelling evidence of the interaction between neuroinflammation, stress, and mental health (Najjar et al. 2013; Pape et al. 2019). Multiple clinical studies have observed disrupted immune signaling in patients diagnosed with a stress-related mental disorder including microglial activation, increased pro-inflammatory cytokines and chemokines, self-reactive T cells, and disrupted blood–brain barrier, among others (Goldsmith et al. 2016; Hasselmann et al. 2018; Liu et al. 2012; Najjar et al. 2017). Furthermore, psychological stress in healthy human individuals also increases the activity of peripheral monocyte recruitment and central microglial activity (Atanackovic et al. 2006; Gouin et al. 2012; Moieni et al. 2015).

In parallel, evidence from rodent studies has demonstrated that different procedures that induce stress mobilize the innate immune system (Leonard and Song 2002). Multiple studies show that the behavioral outcomes of social stress (increased anxiety, social interaction deficits, depressive-like behavior, anhedonia, enhanced vulnerability to rewarding properties of drugs of abuse) are associated with central inflammatory biomarkers (Ambrée et al. 2018; McKim et al. 2016; Wohleb et al. 2015). More specifically, social defeat (SD) stress promotes the activation of toll-like receptors (TLRs), enhances microglia activity, mobilizes neutrophils and monocytes, increases BBB permeability, and increases the secretion of pro-inflammatory cytokines such as interleukin (IL)-6, IL-10, and IL-1b (Ishikawa et al. 2021; Montagud-Romero et al. 2021; Weber et al. 2016; Zhang et al. 2020). This pro-inflammatory profile has been shown to be an important contributor to the defeated phenotype (Ballestin et al. 2021).

The acknowledgement of a relevant contribution of the immune response to the negative consequences of stress has prompted the testing of pharmacological agents with anti-inflammatory action for the treatment of stress-related mental disorders. Oleoylethanolamide (OEA) is an endogenous lipid-derived messenger belonging to the N-acylethanolamines family (Romano et al. 2015; Thabuis et al. 2008). There are multiple reports of the antidepressant effect of OEA in different animal models of depression (Yu et al. 2015).

OEA has been observed to reverse the behavioral deficits induced by chronic SD in male mice (Rani et al. 2021). In their study, SD occurred daily for a period of 21 days. OEA (10 mg/kg) treatment immediately before SD rescued social-avoidance behavior and cognitive impairment caused by social stress. Similarly, in a previous study, we observed that exogenous administration of OEA in socially defeated mice prevented stress-induced cocaine CPP and counteracted the upregulation of TLR4 signaling induced by SD (González-Portilla et al. 2023).

A large body of research from both human studies and animal models has revealed sex differences in the behavioral and immune components of the stress response (Martinez-Muniz and Wood 2020). Due to these sex-specific mechanisms, it has been proposed that the greater incidence of affective disorders including depression and anxiety among women may be due to an increased susceptibility to the effects of stress-induced inflammation on mood and behavior (Derry et al. 2015; Seney & Sibille 2014). Surprisingly, most research studying the neuroimmune consequences of social stress continues to be conducted only on male subjects (Bekhbat and Neigh 2018).

The SD paradigm provides an ethologically significant model for studying the effects of psychosocial stress in rodents (Miczek et al. 2008). SD occurs as a result of experiencing an agonistic encounter with a dominant conspecific animal. Although there are numerous protocol variations, at its core, the experimental mouse (intruder) is placed into the home cage of an aggressive opponent (resident) who will attack the intruder until there is a clear display of submissive behavior. Since the SD model relies on the natural territorial aggression that occurs between adult males, the inclusion of female mice has represented a challenge for the study of SD stress. Several attempts have been made to adapt the SD protocol for achieving a female counterpart of the physiological and behavioral phenotype observed in defeated male mice (Harris et al. 2017; Newman et al. 2019; Takahashi et al. 2017). Vicarious SD (VSD) protocols’ strategy consists of placing the experimental female mouse in an adjacent compartment from where it witnesses the agonistic encounter between two males. The VSD experience has been proven to be a potent stressor that induces in female mice the behavioral outcomes of classic SD in male mice (Carnevali et al. 2020; Iñiguez et al. 2018; Ródenas-González et al. 2023; Warren et al. 2020).

The aim of this study was to evaluate the effect of OEA on behavioral alterations induced by social stress using the SD and VSD models in male and female mice, respectively. Additionally, we evaluated the effect of OEA on the SD-induced inflammatory profile by measuring IL-6 and CX3CL1 (fractalkine) levels in two brain regions, the striatum and hippocampus.

## Methods and materials

### Experimental design

**Fig. 1:**
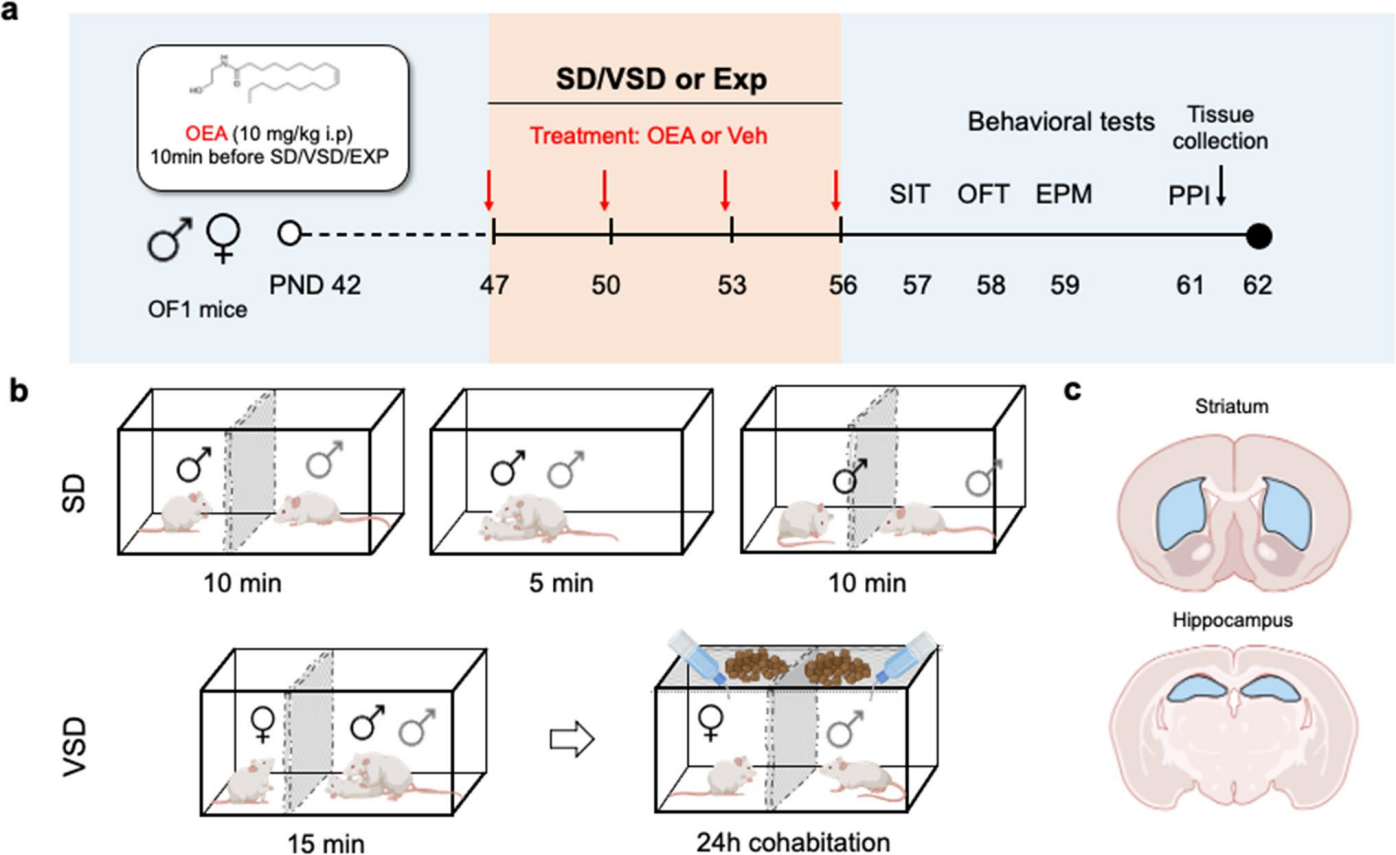
Experimental design. (**a**) Timeline of the experimental design. (**b**) Social defeat (SD) procedure in male mice and vicarious social defeat (VSD) procedure in female mice. (**c**) Diagram of the brain regions dissected for immunoassay analysis

Male and female OF1 mice (*n* = 56) were used as experimental animals (Charles River, France). On arrival, mice were housed in standard cages in a controlled temperature and humidity under a 12 h light/dark cycle with ad libitum access to food and water. Mice were randomly assigned to a stress condition (EXP or SD) and treatment condition (vehicle or OEA) (Fig. 1a).

A set of male OF1 mice (*n* = 15) were used as intruders for the VSD procedure. OF1 male mice used as aggressive opponents (*n* = 20) were housed individually for a month prior to the experiment to heighten territorial aggression. All behavioral testing was conducted during the light cycle.

All procedures were conducted in compliance with the guidelines of the European Council Directive 2010/63/UE regulating animal research and were approved by the local ethics committees of the University of Valencia (2022 VSC PEA 0002).

### Social defeat stress

#### Social defeat stress in male mice

The SD protocol was performed in this study as described in previous works (Montagud-Romero et al. 2017). Mice in the stress condition were exposed to four episodes of SD (Fig. 1b). Each of the SD encounters lasted 25 min and consisted of three phases, each of which began by introducing the experimental animal into the home cage of the “resident” (a male aggressive opponent). During this first phase, the experimental animal is protected from the resident by a wire mesh allowing for social interaction and facilitating instigation and provocation. After 10 min, the wire mesh is removed to allow confrontation between the two animals for a video-recorded 5-min period. In the third phase, the wire mesh is set again for a further 10 min (Rodriguez-Arias et al. 2017).

The non-stressed exploration (EXP) group underwent the same protocol but without the presence of a “resident” opponent in the cage.

#### Vicarious social defeat stress in female mice

The VSD paradigm was performed based on the previously described (Iñiguez et al. 2018; Ródenas-González et al. 2023). Female OF1 mice vicariously experience the defeat of a male OF1 counterpart. In this protocol, VSD females were exposed to non-physical sensory stimuli (visual, olfactory, and chemosensory) associated with indirectly experiencing the defeat of the physically stressed male mouse. For each SD session (15 min/day), intruder male mice were placed into the same compartment as the aggressive resident, while VSD female mice were placed in the neighboring compartment, allowing only a vicarious experience (i.e., visual, olfactory, auditory) of the aggressive encounter. Females were exposed to four episodes of SD vicariously, and following each session, the female mouse stayed housed for 24 h with the resident, separated from the aggressive male mice with a perforated Plexiglas wall (31 × 18 × 0.6 cm) in between both areas (Fig. 1b). Females were physically protected from the male encounter but not from visual, olfactory, and auditory threats, which are part of the vicarious episode. After 24 h, the female was taken back to her home cage and their mates until the following encounter. The female control group (EXP) underwent the same protocol, but without the presence of a male SD encounter in the cage and without the presence of the resident mouse during the 24 h of housing.

### Drug administration

OEA (10 mg/kg, i.p.; synthesized as described by Rodríguez De Fonseca et al. (2001)) was dissolved in 5% Tween 80 in saline and injected 10 min before the corresponding testing of interest. The doses were chosen according to previous studies in rodents reporting effective anti-inflammatory effects (González-Portilla et al. 2023; Moya et al. 2021; Rivera et al. 2018; Sayd et al. 2015).

### Assessment of depressive and anxiety-like behaviors

#### Social interaction test (SIT)

Mice were habituated to a quiet, dimly lit room 1 h before the test. During the test, each animal was placed in the center of a square arena (white Plexiglas open field, 30 cm on each side and 35 cm high) and its behavior was monitored by video (EthoVision XT 11, 50 fps). Animals were allowed to freely explore the arena twice, for 600 s in each session, during two different experimental sessions. In the first session (object session), an empty perforated Plexiglas cage (10 × 6.5 × 35 cm) was placed in the middle of one wall of the arena. In the second session (social session), an unfamiliar OF1 male mouse was introduced into the cage as a social stimulus. Before each session, the arena was cleaned to minimize odor cues. Between sessions, the experimental mouse was removed from the arena and returned to its home cage for 2 min.

Locomotion and arena occupancy during object and social sessions were determined using the animals’ horizontal position, determined by video tracking software (EthoVision XT 11, Noldus). Measures of arena occupancy, such as time spent in the interaction zone and corners, were quantified. The former is commonly used as a social preference-avoidance score and is calculated by measuring the time spent in a 6.5-cm wide corridor surrounding the restrain cage. Corners were defined as two squares of equal areas on the opposite wall of the arena. The social interaction ratio is calculated by considering the time spent by an experimental mouse in the interaction zone when a social target is presently divided by the time it spends in the interaction zone when the target is absent. A ratio equal to 1 means that equal time has been spent in the presence versus absence of a social target.

#### Elevated plus maze (EPM)

The maze consisted of two open arms (30 × 5 × 0.25 cm) and two enclosed arms (30 × 5 × 15 cm), and a central platform (5 × 5 cm) elevated 45 cm above floor level. The floor of the maze was made of black Plexiglas, and the walls of the enclosed arms were made of clear Plexiglas. Mice were habituated to the room for 1 h prior to testing. At the beginning of each trial, experimental mice were placed on the central platform facing an open arm and were allowed to explore for 5 min. The behavior displayed by the mice during the test was recorded by an automated tracking system (EthoVision XT 11, RRiD: SCR_000441). The number of entries and time and percentage of time spent in each section of the apparatus (open arms, closed arms, central platform) was tracked. The time and percentage of time spent in the open arms and the number of open arm entries are generally used to characterize the anxiolytic effects of drugs. In addition, the number of closed and total entries indicates motor activity.

#### Open-field behavior (OF)

The OF test was used to identify the possible effects of OEA on spontaneous activity. Mice were placed in an open-field dark cage (30.5 × 29 × 35 cm) and were allowed to explore freely for 30 min. The activity was tracked and analyzed using the EthoVision XT software (Noldus Information Technology, Wageningen, the Netherlands, http://www.noldus.com) to determine the total distance traveled, the speed, and the time spent in the center of the cage.

### Prepulse inhibition (PPI)

#### Apparatus

Two startle measuring devices were used, consisting of perforated Plexiglas cylinders (28 × 15 × 17 cm) on top of a platform with a force sensor attached to the floor. The value used in the study is the peak value of the startle response. This value is transduced by an accelerometer; the signal is collected, transduced, and digitized on the computer. The apparatus (mod startle response CERS) and program were purchased from CIBERTEC, S.A, Madrid. Spain.

#### Procedure

Based on Valsamis and Schmid’s previous work, the experiment was carried out in two sessions. On the acclimation day, mice were placed into the holder for 5 min with a constant 65-dB white background noise. On the testing day, the PPI was evaluated in a 45-min-long session. Experimental testing comprised three blocks: (1) acclimation period for 5 min; (2) startle habituation, 50 trials of pulses of 120 dB; and (3) PPI testing, two different prepulse intensities (75 dB and 85 dB during 4 ms each) were used with two different inter-stimulus intervals (30 ms and 100 ms) and one single pulse at an intensity of 120 dB during 20 ms each to calculate the baseline startle. Thus, there were four types of prepulse trials: 75 dB/30 ms, 75 dB/100 ms, 85 dB/30 ms, and 85 dB/100 ms, all followed by a 120-dB pulse. The four types of trials were run 10 times alongside single instances of the 75-, 85-, and 120-dB tones, each in pseudorandom order, totalling 70 trials with a 20-s duration for each trial. The prepulse-alone trials (75/85 dB) were introduced to verify that they were not acting as pulses and to confirm that only the 120-dB pulse was the main stimulus to induce a startle response.

### Tissue sampling for biochemical analyses

Mice were sacrificed by cervical dislocation. Within 2 min, the brain was placed in an ice-cold plate and frozen on dry ice and stored at − 80 °C. The striatum and hippocampus were precisely dissected using a coronal brain matrix.

### IL-6 and CX3CL1 measurements

Frozen brain striatal nuclei were homogenized in 250 mg of tissue/0.5 ml of cold lysis buffer (1% NP-40, 20 mM Tris–HCl pH 8, 130 mM NaCl, 10 mM NaF, 10 μg/ml aprotinin, 10 μg/ml leupeptin, 40 mM DTT, 1 mM Na3VO4, and 10 mM PMSF). Brain homogenates were kept on ice for 30 min and centrifuged at maximum speed for 15 min after being determined by the Bradford assay from ThermoFisher (Ref:23,227). Striatal IL-6 and CX3CL1 concentrations were quantified by using an enzyme-linked immunosorbent assay (Mouse IL-6 ELISA Kit, ab 100,712; Mouse Fractalkine ELISA Kit, ab100683) following the manufacturer’s protocol (Abcam, UK). To determine absorbance, we employed an iMark microplate reader (Bio-RAD) controlled by Microplate Manager 6.2 software. The optical density was read at 450 nm, and the final results were calculated using a standard curve carried according to the manufacturer’s instructions. The data were expressed as pg/mg for tissue samples.

### Statistics

For the analysis of the behavioral tests (EPM, OF, SIT, and PPI), a three-way ANOVA with three between-subject variables—stress (EXP and SD), sex (male, female), and treatment (vehicle, OEA)—was performed. Post-hoc comparisons were accomplished by means of Bonferroni tests. Results are expressed as the mean ± SEM, and statistical significance was set at *p* < 0.05.

The PPI was calculated as a percentage score: PPI (%) = 100 − (startle response for pulse with prepulse × 100/startle response for pulse alone). Statistical analyses were performed using SPSS Statistics v23.

## Results

### Change in body weight

**Fig. 2:**
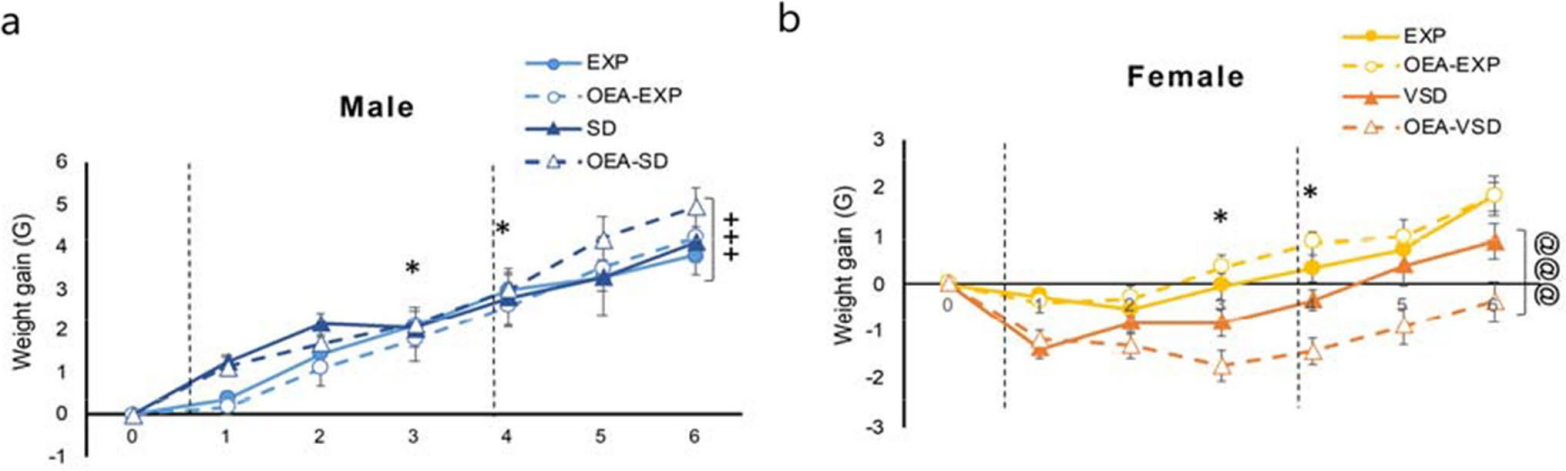
Weight gain of (**a**) male and (**b**) female mice throughout the experiment. The dots represent means and the vertical lines ± SEM of gain weight (gr). Gray zones represent the time span of the SD and VSD protocols. *p < 0.05; **p < 0.01; significant difference of SD/VSD mice with respect to EXP mice. +  +  + *p* < 0.001; significant difference of stressed male mice with respect to female mice; @@@*p* < 0.001 significant difference of VSD female mice compared to EXP female mice

The ANOVA for the weight gain showed an effect of the interaction sex × stress [*F*(1,104) = 14.029, *p* < 0.001]. Vicarious socially defeated female mice decreased weight gain compared to EXP female mice (*p* < 0.001). The ANOVA also revealed a significant effect of the variable day [*F*(5,520) = 140.087, *p* = 0.001], and the interaction day × sex [*F*(5,520) = 15.683, *p* = 0.001], day × stress [*F*(5,520) = 3.243, *p* = 0.007], and day × sex × treatment [*F*(5,520) = 3.262, *p* = 0.003], where OEA-treated VSD female had a marked difference on weight gain. Although weight increased in all mice throughout the experiment (*p* < 0.001), in each day, male mice gain weight was higher compared to female mice (*p* < 0.01). In addition, weight gain in EXP mice was higher on days 3 and 4 (*p* = 0.01) compared to defeated mice, mostly due to stressed female groups (Fig. 2).

### OEA sex-dependent effects on anxiety and depressive-like behavior induced by social stress

#### Social interaction test

**Fig. 3:**
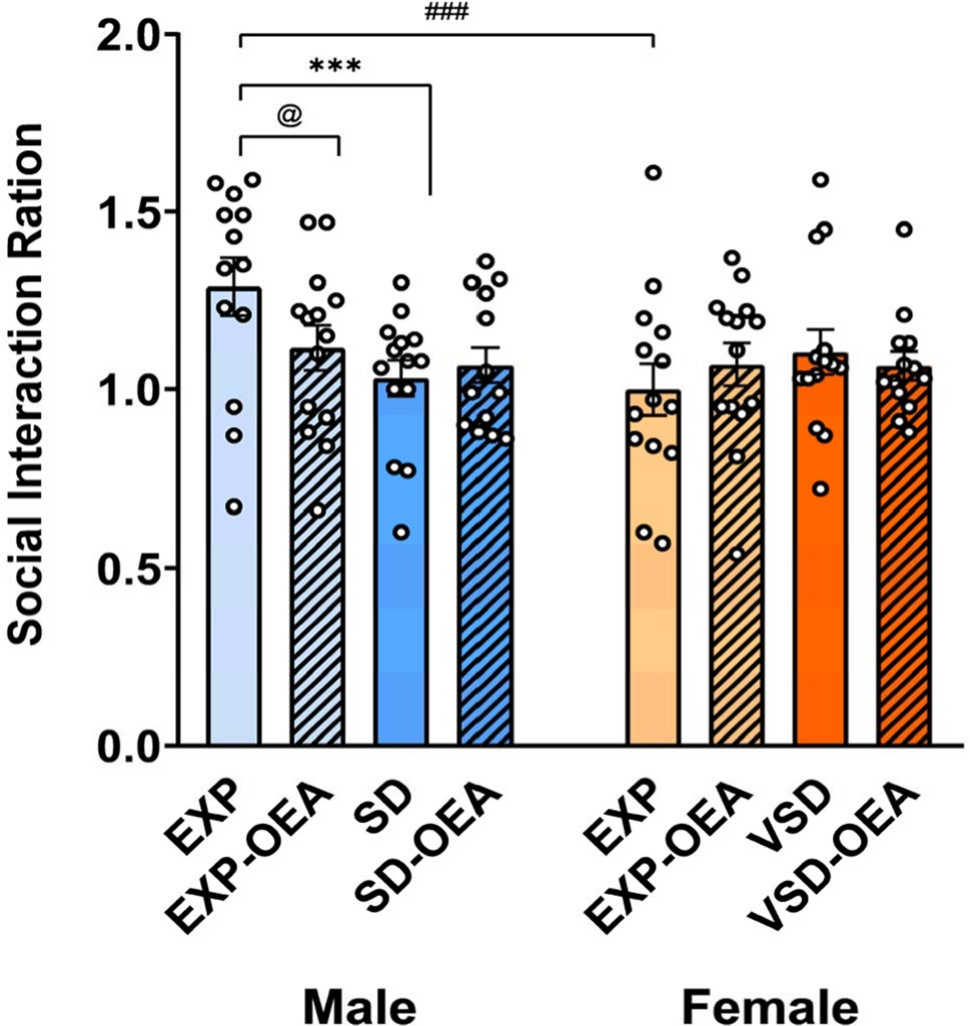
Effects of SD, VSD, and OEA treatment on social interaction. Data are presented as mean values (± SEM). ***p < 0.001, significant difference of SD group with respect to the EXP group; ###*p* < 0.001, significant difference of male EXP mice with respect to female EXP mice; @*p* < 0.05, significant difference of EXP-OEA male mice with respect to EXP male mice

The ANOVA of the SIT revealed an effect of the interaction “sex × stress × treatment” [*F*(1,3604) = 5,160; *p* = 0.002]. Non-stressed vehicle-treated (EXP) male mice exhibited increased social interaction compared to male SD mice (*p* < 0.001). Equally, EXP male mice exhibited increased social interaction compared to EXP female mice (*p* < 0.001) and EXP-OEA male mice (*p* = 0.011) (Fig. 3).

#### Open field

**Fig. 4:**
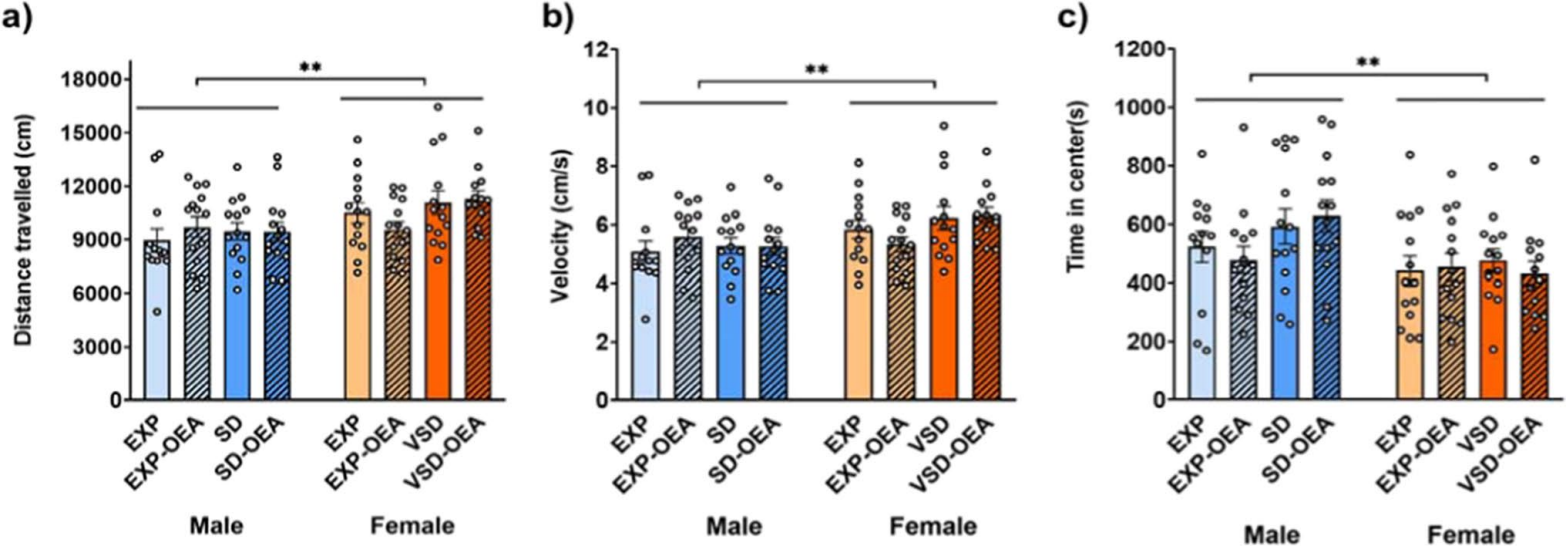
Effects of SD, VSD, and OEA treatment on the OF test. (**a**) Distance traveled; (**b**) velocity; (**c**) time in center. Data are presented as mean values (± SEM). **p < 0.01, significant difference with respect to female mice

In the OF test, we found an effect of the variable “sex” for the distance traveled [*F*(1,102) = 9,337; *p* = 0.003], velocity [(1,102) = 8,287; *p* = 0.005] and time in the center [*F*(1,102) = 8,773; *p* = 0.004]. Female mice traveled a greater distance and at a higher speed compared to male mice. However, female mice spent less time in the center zone compared to male mice (Fig. 4). As expected, OEA-treated mice did not show any significant differences in OF behavior with respect to the vehicle-treated groups.

#### Elevated plus maze

The ANOVA of the EPM test is presented in Table 1. The ANOVA revealed an effect of the interaction stress × treatment for the time spent in open arms [*F*(1,91) = 5,057; *p* = 0.027] and the number of open entries [*F*(1,91) = 6,435; *p* = 0.013]. In vehicle-treated mice, defeated mice spent less time in the open arms (*p* < 0.001), an effect also presented in the defeated groups treated with OEA (*p* < 0.05). However, in both defeated male and female mice, OEA treatment increased the number of open entries (*p* < 0.01).

**Table 1:** Effects of SD, VSD, and OEA treatment on the elevated plus maze (*n* =  ± .12 per group)

	EXP	EXP-OEA	VSD	VSD-OEA
Male mice				
Time in OA	76 ± 9	58 ± 7	44 ± 8***	46 ± 9*
Time in CA	112 ± 9	122 ± 11	121 ± 8	119 ± 7
% time OA	40 ± 4	33 ± 4	26 ± 4	26 ± 5
Open entries	14 ± 1	12 ± 1	12 ± 1	14 ± 1**
% Open entries	43 ± 2	40 ± 4	33 ± 4***	32 ± 3***
Total entries	33 ± 2	31 ± 2	36 ± 1	43 ± 1 +
Female mice				
Time in OA	76 ± 11	58 ± 9	43 ± 7***	59 ± 6*
Time in CA	111 ± 10	131 ± 14	147 ± 8**	154 ± 8**
% time OA	40 ± 4	31 ± 5	22 ± 3	27 ± 2
Open entries	18 ± 3	12 ± 1	17 ± 2	21 ± 1**
% Open entries	42 ± 5	31 ± 3	60 ± 4***	68 ± 3***
Total entries	41 ± 3	37 ± 1	27 ± 2	31 ± 1 +

Data are presented as mean values (± SEM)

^*^p < 0.05, significant difference with respect to the corresponding EXP and EXP-OEA mice

^**^p < 0.01, significant difference with respect to the corresponding EXP and EXP-OEA mice

^***^p < 0.001, significant difference with respect to the corresponding EXP and EXP-OEA mice

+ *p* < 0.05, significant difference with respect to the corresponding vehicle-treated group

The ANOVA also revealed an effect of the interaction sex × stress in time spent in the closed arms [*F*(1,91) = 4,254; *p* = 0.042] and the percentage of open entries [*F*(1,91) = 42,307; *p* < 0.001]. VSD female spent more time in closed arms than non-stressed female (*p* < 0.01) and defeated male mice (*p* < 0.001). Defeated male mice decreased the percentage of open entries when compared with non-stressed mice (*p* < 0.001), although the opposite effect was observed in VSD female mice (*p* < 0.001). In addition, VSD female mice made a higher percentage of open entries compared to SD male mice (*p* < 0.001).

For the number of total entries, the ANOVA revealed an effect of the interaction sex × stress [*F*(1,91) = 29,175; *p* < 0.001] and stress × treatment [*F*(1,91) = 7,394; *p* = 0.008]. Although non-stressed female mice made a higher number of total entries than males, converse results were observed in defeated mice (*p* < 0.05). In stressed mice, a higher number of total entries were observed in OEA-treated mice with respect to vehicle-treated mice (*p* < 0.05). In addition, in vehicle-treated mice, EXP mice exhibited a higher number of total entries with respect to the SD group (*p* < 0.05).

### OEA restores PPI deficits induced by social stress

**Fig. 5:**
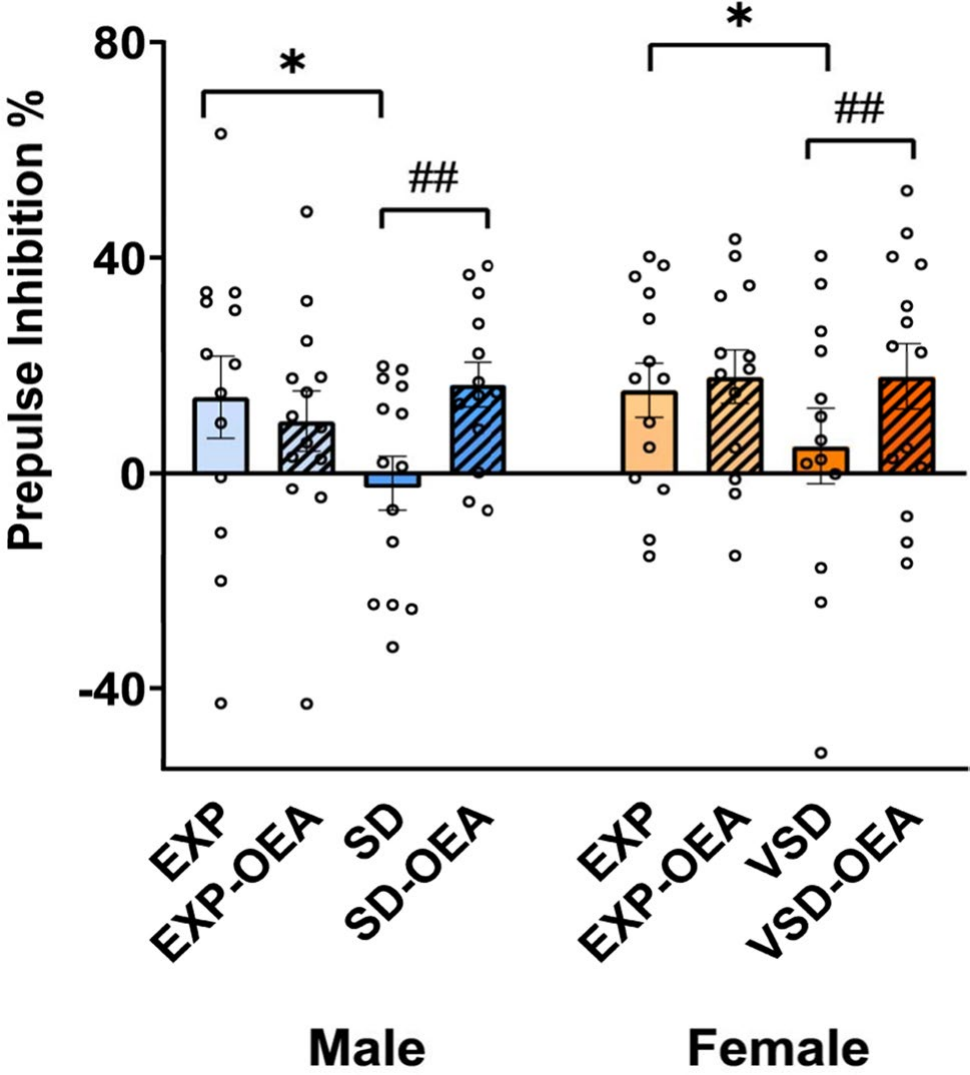
Effects of SD, VSD, and OEA treatment on prepulse inhibition. Data are presented as mean values (± SEM). *p < 0.05, significant difference of SD mice with respect to EXP mice (both male and female); ##*p* < 0.01, significant difference of SD-OEA group with respect to SD group (both male and female)

The ANOVA of the PPI revealed an effect of the interaction “stress × treatment” [*F*(1,100) = 4,159; *p* = 0.044]. Vehicle-treated EXP mice showed increased PPI compared to SD mice (*p* = 0.024). In addition, SD/VSD mice that received OEA treatment exhibited an increased PPI compared to vehicle-treated SD/VSD mice (*p* = 0.008) (Fig. 5).

### Divergent neuroinflammatory profile induced by SD and VSD in male and female mice

The ANOVA for the CX3CL1 levels in the striatum revealed an effect of the interaction sex × stress [*F*(1,68) = 8.026; *p* = 0.006]. EXP male mice exhibited greater detection of CX3CL1 compared to EXP female mice (*p* < 0.001). A higher concentration of CX3CL1 was observed in female mice exposed to VSD compared to EXP female mice (*p* = 0.01).

**Fig. 6:**
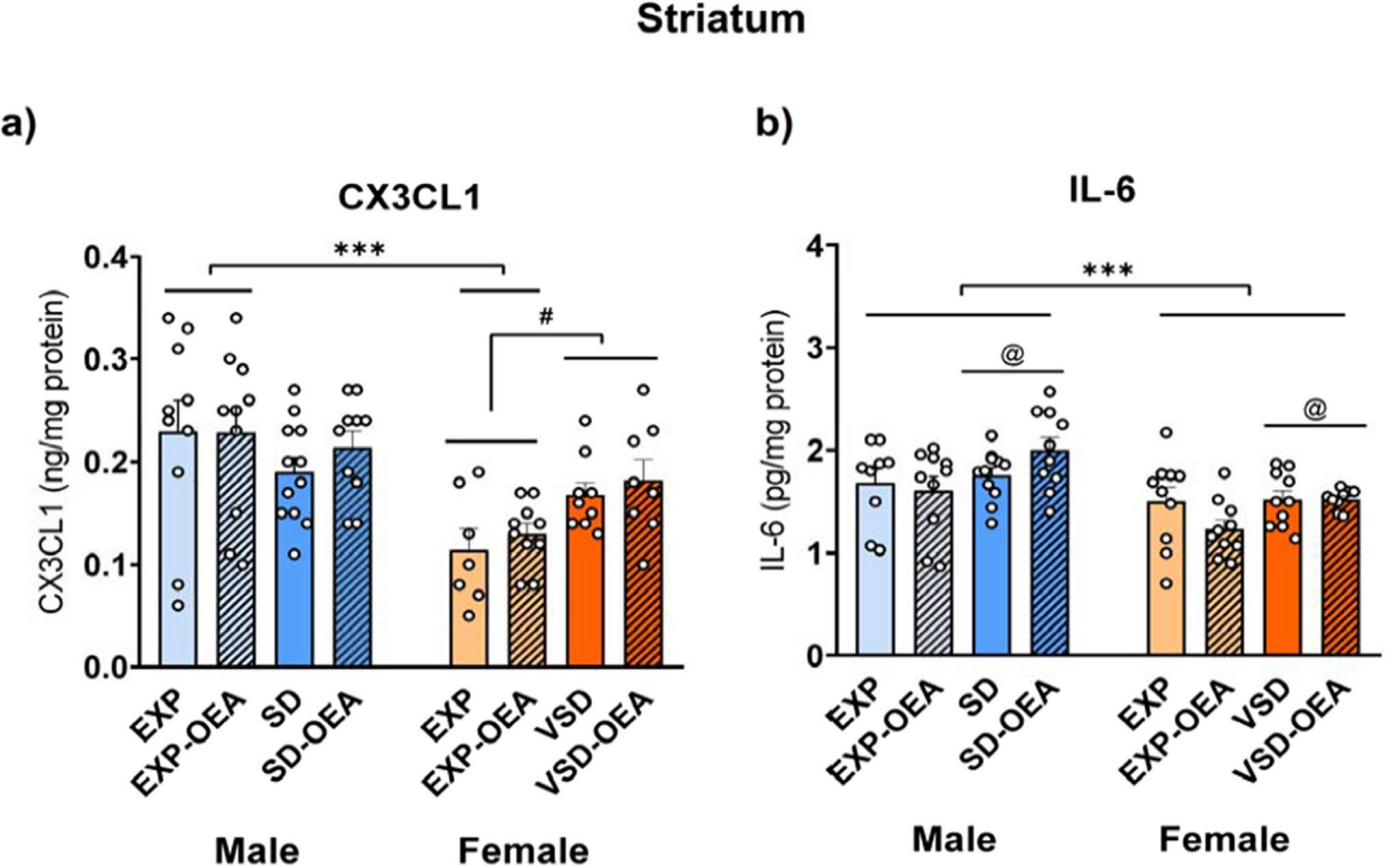
Effects of SD, VSD, and OEA treatment on IL-6 and CX3CL1 striatal levels. Bars represent the mean (± SEM) of the striatal levels (in pg/mg) of (**a**) the pro-inflammatory cytokine IL-6 and (**b**) chemokine CX3CL1 levels (in ng/mg). ***p < 0.001, significant difference of male mice with respect to the corresponding female mice. #*p* < 0.05, significant difference of female EXP mice with respect to female SD mice; @*p* < 0.05, significant difference of stressed mice (SD/VSD) with respect to EXP mice

IL-6 levels in the striatum showed a significant effect of the variable sex [*F*(1,72) = 19.155; *p* < 0.001] and stress [*F*(1,72) = 5.471; *p* < 0.022]. A higher concentration of IL-6 was observed in the striatum of male mice compared to female (*p* < 0.001). Overall, stressed male or female mice exhibited higher levels of IL-6 compared to EXP mice (*p* < 0.05) (Fig. 6).

**Fig. 7:**
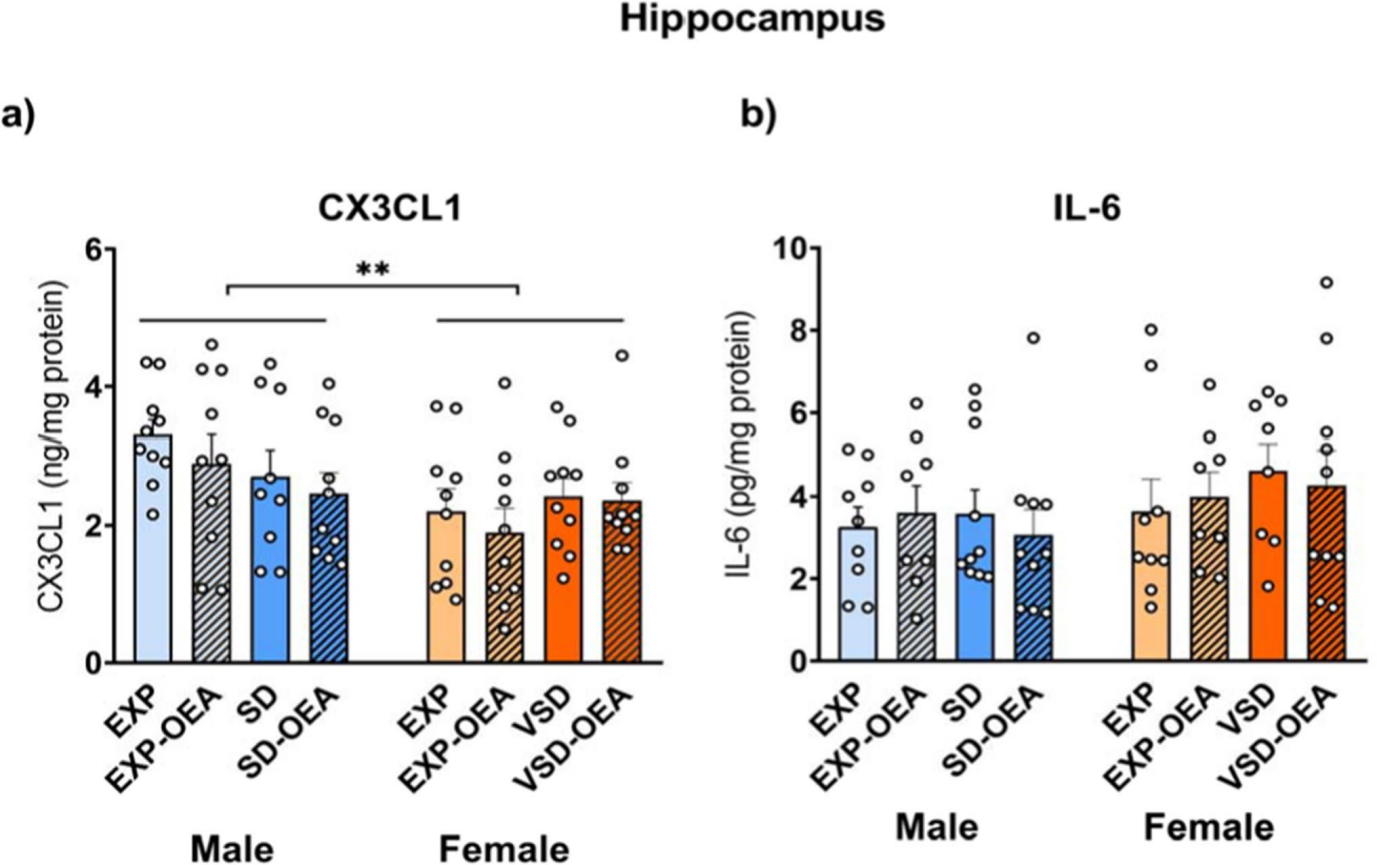
Effects of SD, VSD, and OEA treatment on IL-6 and CX3CL1 hippocampal levels. Bars represent the mean (± SEM) of the hippocampal levels (in pg/mg) of (**a**) the pro-inflammatory cytokine IL-6 and (**b**) chemokine CX3CL1 levels (in ng/mg). **p < 0.01, significant difference of male mice with respect to female mice

The ANOVA of CX3CL1 levels in the hippocampus revealed an effect of the variable sex [*F*(1,71) = 7,434; *p* < 0.008]. Male mice exhibited greater detection of CX3CL1 compared to female mice (*p* < 0.01). No differences were observed in the levels of IL-6 in the hippocampus (Fig. 7).

## Discussion

There are a variety of mental disorders in which stress plays a major role as a risk factor, a trigger, or an aggravating event (Zefferino et al. 2021). The lack of animal models of social stress in female mice has hindered its research. In the present study, we demonstrated that SD and VSD induce maladaptive behavioral impairments and disrupt PPI in male and female mice, a behavioral model of analysis of sensorimotor gating often disrupted in neurodevelopmental disorders such as schizophrenia. Most importantly, we found that OEA treatment restores stress-induced PPI deficits. Also, we observed that these behavioral deficits are accompanied by sex-specific inflammatory signaling changes (IL-6 and CX3CL1) in the striatum and the hippocampus. These results further validate VSD as an effective paradigm of social stress.

### SD and VSD induced behavioral alterations associated with sex-specific pro-inflammatory signaling in the striatum and hippocampus

A primary goal of this study was to evaluate the SD and VSD procedures and, most importantly, the potential differences in the resulting defeated phenotype of male and female mice. SD is a valid paradigm to study the effects of social stress on vulnerability to develop mental disorders (Nasca et al. 2019). Its outcomes in mice resemble those of social isolation: depression, anxiety, and PPI disruption (Ago et al. 2014). Although results should be considered cautiously, the SD is a highly translatable experience that resembles in some aspects to social human conflict, which is one of the main sources of stress in modern society. A large body of evidence proves that mice exposed to SD, as humans exposed to stressful experiences, present an increased risk of developing anxiety-like behavior and behavioral alterations such as social withdrawal and anhedonia, which are core features of depression (Montagud-Romero et al. 2015, 2018; Reguilón et al. 2022).

In this study, we employed VSD, a procedure in which the female experimental mice witness an agonistic encounter between two unfamiliar conspecifics. After VSD, female mice were housed with the resident mice separated by a perforated transparent Plexiglas during 24 h. The VSD procedure has been shown to induce a neuroendocrine stress response in both male and female mice. VSD stimulates corticotropin-releasing hormone release and induces a behavioral phenotype similar to that of male defeated mice (Iñiguez et al. 2018; Ródenas-González et al. 2023; Sial et al. 2016). It is established that repeated exposure to SD induces social withdrawal and increased anxiety-like behavior (Giménez-Gómez et al. 2021; Macedo et al. 2018). Consistent with this, we observed that SD male mice exhibited social withdrawal as they spent less time in the interaction zone compared to EXP male mice. On the contrary, VSD did not induce social avoidance, as female mice across experimental conditions displayed similar SI ratios. This is consistent with previous works that show that social stress in females does not affect social interaction behavior (Ródenas-González et al. 2023). These results suggest that although witnessing an aggressive SD encounter is perceived as stressful, female mice do not find interaction with the resident mice aversive or threatening. A plausible explanation for the low percentage of stressed females showing social avoidance is a confounding increased sexual motivation. It has been reported that female mice exhibit a socio-sexual preference toward dominant mice (Parmigiani et al. 2009; Rich and Hurst 1998). It is possible that witnessing and being exposed to the chemosensory signals of a dominant male during cohabitation increases sexual motivation (Haga et al. 2010; Moncho-Bogani et al. 2002). It is important to remark that inter-male territorial aggression, as occurs in SD, is a testosterone-dependent behavior with an ethological relevance on mice mating behavior.

Furthermore, our results showed that SD induced an increase in anxiety-like behavior. As repeatedly reported, SD male mice decreased the time spent in the open arms of the EPM compared to non-stressed mice. A similar effect was observed in female mice, in agreement with our previous studies (Ródenas-González et al. 2023; Yohn et al. 2019). However, while stressed male mice made more total entries and decreased the percentage of open entries, VSD female made fewer number of total entries but increased the percentage of open entries. Therefore, the anxiogenic profile induced by social stress showed sex-dependent particularities. In this line, although we did not find any differences between control and stressed mice in the OF test, female mice showed increased distance traveled, velocity and time in the center, according to their baseline greater novelty-induced anxiety compared to male mice (Johnston and File 1991; Miller et al. 2021).

In agreement with previous studies, we observed a reduction in weight gain as a result of vicarious stress exposure (Sial et al. 2016; Iñiguez et al. 2018). Female stressed mice suffered a significant reduction of gain weight compared to EXP mice, especially at the end of the VSD procedure (days 3 and 4). Compared to SD male mice, the reduction in gain weight in VSD female mice may be more prominent as a result of the 24 h cohabitation with a male resident, in which the source of stress was maintained and thus could affect activity and food intake. In summary, our results highlight the importance of design elements and sex differences in the behavioral outcomes of SD and VSD.

It is important to assess the ecological validity of the social defeat experience. Territorial aggression is a naturally occurring interaction between male mice in proximity. A sexually mature male will attack an individual that enters his territory mainly by relying on chemosensory signals. However, it is important to remark that in naturalistic conditions, defeated mice would have the ability to avoid or escape from potential aggressive opponents. Also, defeated mice adopt a submissive posture that prevents further instigation and attacks from a dominant male. Forced repeated exposures to dyadic defeat with different resident mice in a closed time window does not contribute to the ecological validity of the model (Lyons et al. 2023).

The VSD has been proposed as a suitable stress model for studying stress in female mice. One of the strengths of the VSD model is that, since direct physical interaction is prevented by a physical barrier, the nature of stress is purely psychological. This variation improves the face validity of VSD with respect to the standard SD as a procedure to study the consequences of psychosocial stress. Nevertheless, we acknowledge that the continuous sensory but not physical contact during the 24-h-long cohabitation with the resident mice in VSD female mice is not a representative experience of naturalistic conditions (Lyons et al. 2023). In this sense, some of the design elements of the VSD could differentially affect the defeated phenotype.

The immune response has been shown to be an important contributor to the severity of depressive-like and anxiety behavior induced by SD (Montagud-Romero et al. 2021). Our results show that both SD and VSD have negative behavioral effects which correlate with increased pro-inflammatory signaling. Overall, we found increased striatal IL-6 and hippocampal CX3CL1 protein detection in males compared to females.

To our knowledge, no previous reports have observed these differences. However, the sexual disparity in the production of cytokines in response to stress has been previously reported in both humans and rodents (Bekhbat and Neigh 2018). It has been shown that although females exhibit decreased release of the cytokine in response to stress, they may be more vulnerable to their effects on behavior (Derry et al. 2015; Medina-Rodriguez et al. 2022). The increase in neuroinflammatory markers in non-stressed male mice could be due to the higher social conflict in their home cage compared to female mice.

In rodent models, IL-6 plays a crucial role in the affective and cognitive impairments resulting from social stress (Chourbaji et al. 2006; Niraula et al. 2019). In this study, biochemical analysis showed that both male and female stressed mice exhibited increased striatal IL-6 levels in the striatum. These results are consistent with previous studies showing an increase in IL-6 levels after SD in male mice (Ferrer-Pérez et al. 2019; Reguilón et al. 2022). In addition, this data is in agreement with the study of Takahashi et al. (2017), which also found stressed-induced increased levels of IL-6 in stressed female mice compared to control mice. On the contrary, we previously reported no differences in IL-6 levels in the striatum of female mice immediately after the last VSD (Ródenas-Gonzalez et al. 2023). Nevertheless, differences in the timing of the measure and the experimental design could explain these differences.

Our laboratory and others have previously described the involvement of the CX3CL1/CX3CR1 signal axis in controlling microglial activation after SD stress exposure (Liu et al. 2020; Montagud-Romero et al. 2020). As an important regulator of microglial activity, CX3CL1 function is crucial for the triggering of secondary cascades that regulate the neuroinflammatory response. We found increased CX3CL1 levels in the hippocampus of stressed female mice. The observed sex-specific effects in hippocampal CX3CL1 levels might be a result of a distinct impact of stress on the immune response that differs between sexes. It is important to remark that ovarian hormones affect stress-induced increases in pro-inflammatory cytokines and chemokines as well as sensitivity to their effects on behavior (Finnell et al. 2018). Another possibility is that the differences in CX3CL1 levels observed in stressed female mice are due to the qualitative difference between direct/physical vs. vicarious/emotional stress in male and female mice exposed to SD and VSD, respectively.

### OEA effects on anxiety and depressive-like behavior induced by SD and VSD

There are multiple reports showing an antidepressant effect of OEA using both physical and psychological stressors (Costa et al. 2018; Rani et al. 2021). A body of research has established a 10 mg/kg OEA dose as achieving the maximum therapeutic effect (Orio et al. 2019). Jin et al. (2015a, b) showed that OEA reversed anhedonia and anxiety-like behavior caused by a chronic unpredictable mild stress protocol. More recently, OEA has been proven to have a potent antidepressant effect on the behavioral alterations induced by SD (González-Portilla et al. 2023; Rani et al. 2021). To the best of our knowledge, this is the first study exploring the effects of OEA on stress-induced behavioral deficits in female mice. In our study, OEA treatment resulted in sex-specific effects on anxiety and depressive-like behavior induced by social stress.

We performed the SIT test 24 h after the last SD/VSD encounter to test whether OEA treatment could rescue stress-induced social withdrawal. In our study, only defeated male mice showed a decrease in social interaction without a protective effect of OEA. A previous study reported that daily treatment with OEA (10 mg/kg) prevented social interaction deficits caused by chronic SD (Rani et al. 2021). In this sense, the longer duration of the stress procedure (21 days) and, accordingly, the greater number of OEA doses administered could explain the absence of an effect of OEA on SI in our experimental procedure.

Previous studies reported that OEA (1 mg/kg, 5 mg/kg, and 10 mg/kg) did not increase anxious behavior in the EPM (Campolongo et al. 2009; Joshi et al. 2018). Here, OEA did not affect overall performance on the EPM test in non-stressed mice. While time in open arms was not restored by OEA administration, an increase in the number of open entries was observed in OEA-stressed mice, suggesting an anxiolytic effect.

Given the observed anti-inflammatory effects of OEA, we hypothesized that OEA treatment could attenuate the pro-inflammatory cascade induced by SD. Nevertheless, we did not observe differences between vehicle-treated and OEA-treated mice in the striatal and hippocampal IL-6 and CX3CL1 levels. We argue that the tissue sample collection was too distant from the stress exposure for detecting OEA effects on SD-induced inflammation. This difference in the timing of brain tissue collection could explain the lack of differences between vehicle and OEA-treated mice. Previous reports on the anti-inflammatory effects of OEA found a reduction of inflammatory biomarkers (IL-1β, IL-6, interferon-gamma-γ, CCL2, tumor necrosis factor-α) when measured immediately after an immune challenging event (Joshi et al. 2018; Sayd et al. 2015). After one week, it is possible that the anti-inflammatory effects of OEA on SD-induced immune challenge were no longer detectable. One limitation of our study is that we only assessed the effects of a single dose of OEA (10 mg/kg). In this sense, it is not possible to discard dose-dependent effects or that a higher dose of OEA could have resulted in more prominent effects on behavior and inflammatory signaling.

### OEA restores stress-induced-lowered PPI in both male and female mice

PPI of the acoustic startle reflex is used to assess basic sensorimotor gating mechanisms (Geyer et al. 2002). Performance in the PPI test is primarily associated with the functioning of the dopaminergic system (Koch 1999; Schmajuk et al. 2009). Previous studies show that endocrine responses to stress such as prolonged elevation in corticosterone levels can affect dopamine-dependent sensorimotor gating in mice (Conti et al. 2002). In this regard, striatal dopamine dysfunction is involved in multiple behavioral changes reported after SD stress including social withdrawal, anhedonia, and the potentiation of rewarding properties of drugs of abuse (Huang et al. 2016; Jin et al. 2015a, b; Larrieu et al. 2017; Watt et al. 2014). Several studies have demonstrated that repeated SD exposure leads to mesolimbic sensitization (Montagud-Romero et al. 2017; Tidey and Miczek 1996), increasing baseline dopaminergic neurotransmission (Selten et al. 2013). In parallel, several studies have shown that repeated SD can lead to PPI deficits (Adamcio et al. 2009; Schnider et al. 2022).

In this study, we observed that SD and VSD decreased PPI in both male and female mice. Most notably, 10 mg/kg OEA administration before each SD encounter was able to prevent these deficits in both male and female mice. In our study, OEA treatment had no effect on the long-term increased pro-inflammatory signaling in the striatum and hippocampus but may reverse or alleviate some of the disturbances in dopaminergic signaling caused by SD stress. Although we did not obtain any specific measure of dopamine function, our results suggest that OEA may restore dopaminergic signaling altered by SD. In support of this hypothesis, recent studies suggest a modulatory effect of OEA on the dopamine system. Thus, OEA is able of normalizing striatal dopamine release associated with a high-fat diet, counteracting the anhedonic response to low-calorie meals (Tellez et al. 2013). Additionally, Romano et al. (2020) proved that OEA restores a normal dopamine surge to food stimuli in mice that display stress-induced binge eating. In line with this, our results provide indirect evidence that SD and VSD alter dopaminergic signaling in the striatum and, most importantly, that acute and contingent OEA treatment before SD restores stress-induced PPI alterations. Further studies should investigate the specific mechanisms by which OEA interacts with dopamine signaling in reward-related brain regions. The contribution of other OEA-modulated transmitters involved in PPR, such as histamine or serotonin, cannot be ruled out.

## Conclusions

In this study, we described the behavioral profile resulting from SD and VSD in male and female mice, respectively. We observed that SD and VSD increased anxiety-like behavior, induced social interaction deficits in male mice, and decreased PPI in both male and female mice. As observed in previous studies, we confirmed that SD and VSD induced an increase in pro-inflammatory signaling in the striatum and hippocampus. Most notably, we observed that OEA treatment before SD/VSD attenuated stress-induced PPI alterations in both male and female mice. Together, the OEA sex-dependent effects on anxiety and depressive-like behavior require further research, but our data suggest a therapeutic effect of OEA on stress-induced behavioral alterations.

## Data Availability

The data are available for any scientific use from the corresponding author on reasonable request.

## Abbreviations

CA: Closed arms
CNS: Central nervous system
CPP: Conditioned place preference
ELISA: Enzyme-linked immunosorbent assay
EPM: Elevated plus maze
EXP: Exploration
IL: Interleukin
OA: Open arms
OEA: Oleoylethanolamide
OF: Open field
PND: Postnatal day
PPI: Prepulse inhibition
SD: Social defeat
SIT: Social interaction test
TLR: Toll-like receptor
TRPV1: Transient receptor potential vanilloid 1
VSD: Vicarious social defeat
